# WTAP: a new player in postnatal BAT development

**DOI:** 10.1093/lifemeta/loac031

**Published:** 2022-11-03

**Authors:** Lei Sun

**Affiliations:** Cardiovascular and Metabolic Disorders Program, Duke-NUS Medical School, Singapore 169857, Singapore

**Understanding the mechanisms underlying brown fat development and metabolism can provide unique insights into the regulation of energy homeostasis. In a recent study published in *Life Metabolism*, Wang *et al*. established Wilms’ tumor 1-associating protein (WTAP), a key component in m** ^**6**^ **A methyltransferase complex, as a new and essential regulator in the postnatal development and maturation of interscapular brown adipose tissue (iBAT).**

Interscapular brown adipose tissue (iBAT) is the major organ responsible for nonshivering thermogenesis in rodents [1]. Understanding the basic mechanisms underlying its development and metabolism will provide unique insights into how cells consume a large amount of nutrient fuels rapidly for energy expenditure and how such a drastic process of energy conversion is regulated. These mechanisms can be utilized to improve metabolic health by designing new strategies to remodulate energy homeostasis at the cellular and systemic levels. In the past two decades, multiple factors such as PR domain-containing protein 16 (PRDM16) and peroxisome proliferator-activated receptor gamma (PPARγ) have been identified as regulators for iBAT development [2]. However, the program controlling adipocyte biology does not only involve protein factors but also multiple regulatory layers at RNA processing levels such as N6-methyladenosine (m^6^A) mRNA modifications [3].

m^6^A was originally discovered in 1970s but only in scattered studies [4], and methyltransferase-like 3 (METTL3) was established as a key player in methyltransferase complex in 1990s [5]. As the next-generation-sequencing technology develops, the past two decades witnessed a surge of research interest in RNA methylation. Currently, we know that m^6^A modification is the most prevalent internal mRNA modification in mammals and occurs at many thousands of sites across the transcriptome, which generates a wide-range of influence on gene expression, stability, and translational efficiency [6]. The biological function of each component of RNA methylation processing machinery—writers, erasers, and readers—is being intensively investigated.

METTL3, the most established m^6^A methyltransferase, was reported to play a key role in postnatal development of iBAT [7]. In a recent study published in *Life Metabolism*, Wang *et al*. described the function of Wilms’ tumor 1-associating protein (WTAP) another essential component of METTL3-containing methyltransferase complex [8], in regulating iBAT development [9]. They found that WTAP protein is significantly enriched in iBAT compared with inguinal white adipose tissue and epididymal white adipose tissue. It is significantly increased during the postnatal development of iBAT, reaching its peak between 10 and 20 days of age, mirroring the pattern of METTL3 [3]. BAT-specific knockout of *Wtap* (*Wtap*-BKO) severely impairs BAT development and decreases the expression of BAT-selective genes including *Prdm16*, *Ucp1*, and *Pgc1α*. *Wtap*-BKO mice fail to maintain their body temperature and succumb to acute cold challenge, demonstrating the necessity of WTAP in maintaining thermogenesis capacity of BAT.

**Figure 1:**
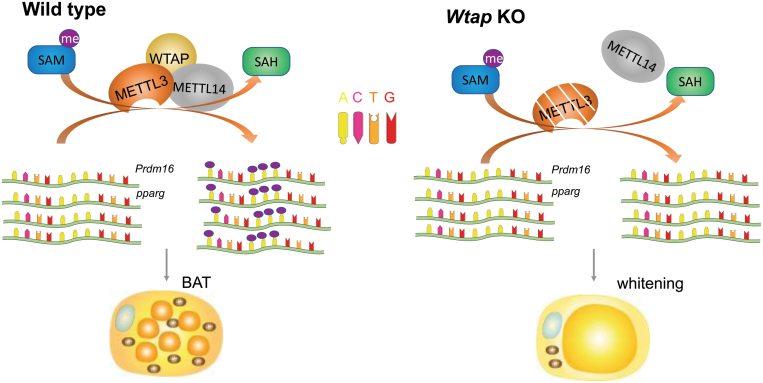
*Wtap* knockout results in an impaired BAT postnatal development and whitening of adult BAT. It does so through destabilizing METTL3 and reducing m^6^A methylation in brown fat transcripts including *Prdm16* and *Pparg* mRNAs.

Mechanistically, WTAP deficiency results in a proteosome-dependent instability of METTL3 and decreases m^6^A mRNA modification in thousands of transcripts. Key players in major BAT metabolism pathways feature in these transcripts, including *Prdm16* and *Pparg* mRNAs (Fig. 1). The widespread decrease of RNA methylation suggests that WTAP should exert its function mainly through the METTL3-mediated mRNA methylation. Indeed, *Mettl3* overexpression in BAT partially rescues the phenotypes in *Wtap*-BKO mice. This work, together with the earlier METTL3 study [3], demonstrates an essential role of WTAP/METTL3-mediated RNA methylation in iBAT development and thermogenesis.

Our understanding about the regulation of RNA methylation in adipocyte development and metabolism is still at infancy stage and many outstanding questions remain to be addressed. Both METTL3 deficiency and WTAP deficiency impair BAT development and thermogenesis [3, 9], demonstrating the necessity of m^6^A RNA methylation in supporting BAT biology. However, the ablation of METTL3 or WTAP causes a genome-wide decrease of RNA methylation in thousands of transcripts. It is unknown whether any specific transcript may play a more important role in the observed phenotypes. It is conceivable that RNA methylation in key BAT regulators such as *Prdm16* and *Pparg* may be of more relevance than others. However, the functional impacts of RNA modifications are also influenced by other factors. For example, some transcripts are modified at a higher frequency than others, so these transcripts tend to be more susceptible to methylation deficiency. Moreover, depending on the positions of modified nucleotides, m^6^A modifications may affect transcripts’ splicing, stability, or translation. These possibilities will need to be carefully examined in a transcript-specific manner. Besides m^6^A modification, more than a hundred of modifications can occur in RNAs, collectively referred to as epitranscriptome [10]. In a broader picture, how different types of modifications can contribute to BAT as well as white fat biology and how they may coordinate with m^6^A modification are still open questions. We are embarking on an exciting journey to understand the interaction between epitranscriptome and adipose biology. The study about WTAP discussed here has laid a foundation for the future investigation.
